# The Microbiota in Children and Adolescents with Asthma

**DOI:** 10.3390/children11101175

**Published:** 2024-09-27

**Authors:** Lucio Casali, Giulia Maria Stella

**Affiliations:** 1 Unit of Respiratory Diseases, University of Perugia, 06121 Perugia, Italy; luciocasali@gmail.com; 2 Department of Internal Medicine and Medical Therapeutics, University of Pavia Medical School, 27100 Pavia, Italy; 3 Unit of Respiratory Diseases, Cardiothoracic and Vascular Department, IRCCS Policlinico San Matteo, 27100 Pavia, Italy

**Keywords:** microbiota, microbiome, bronchial asthma, children, adolescents

## Abstract

The role of the respiratory microbiome has been deeply explored for at least two decades. Its characterization using modern methods is now well-defined, and the impacts of many microorganisms on health and diseases have been elucidated. Moreover, the acquired knowledge in related fields enables patient stratification based on their risk for disease onset, and the microbiome can play a role in defining possible phenotypes. The interplay between the lung and gut microbiomes is crucial in determining the microbial composition and immuno-inflammatory reaction. Asthma is still not a well-defined condition, where hyperreactivity and the immune system play important roles. In this disease, the microbiome is mostly represented by *Proteobacteria*, *Streptococcus*, and *Veillonella*, while *Cytomegalovirus* and *Epstein–Barr* viruses are the most prevalent viruses. A mycobiome may also be present. The passage from infancy to adolescence is examined by evaluating both the clinical picture and its relationship with possible variations of the microbiome and its effects on asthma. Otherwise, asthma is considered a heterogeneous disease that often starts in childhood and follows a particular personalized track, where adolescence plays a pivotal role in future prognosis. Under this point of view, the microbiota, with its possible variations due to many factors, both internal and external, can modify its composition; consequently, its inflammatory action and role in the immunological response has obvious consequences on the clinical conditions.

## 1. Background

According to the last edition of Global Initiative for Asthma (GINA) [1], asthma is defined as “an heterogenous disease, usually characterized by chronic airway inflammation”. Its symptoms are represented by shortness of breath, cough, chest tightness, and wheeze and vary over time [1]. Pulmonary function is marked by the presence of signs of obstruction that are spirometrically measured, which can be pharmacologically or spontaneously reversed; however, over time, these symptoms can become definitive. At the immunological level, the pathogenesis of asthma is mainly based on the role of T-helper cells, distinguished into a Th2-high or Th2-low phenotype [2,3,4]. Exposure to irritants or allergens may increase the presence of symptoms in individuals with asthma. Asthma is also one of the most frequent diseases in childhood, and the interactions between multiple genetic and environmental factors evoke the immunological, clinical, and obstructive signs that characterize the features of this disease [3,4,5]. Asthma seems to be related to many risk factors, such as urban life, exposure to smoke and pollution, excessive weight gain, prematurity, and airway infections, which are mainly of a viral origin. The interplay of these factors explains the heterogeneity of the disease; therefore, a personalized and complete treatment would be necessary [6,7]. This means that clinical phenotypes, largely based on all the commonly observed features, have their importance, and endotypic characterization is particularly crucial [6]. In the natural history of asthma, it has to be stressed that exacerbations can be triggered by exposure to allergens, tobacco smoke, air pollution, or, as already mentioned, viral infections [8]. The severity of these exacerbations is variable, and their outcome is not always predictable, which could be mild and well-controlled at home or very severe with possible admission to emergency department (ED). One of the most important risk factors is observed at the end of puberty, and this means that, in adolescents, the outcome and subsequent prognosis can be worse [9]. In this sense, the prevalence of asthma as a chronic, non-communicable disease is increasing worldwide [1]. In fact, a prevalence of 39–70% has been reported for severe forms of asthma, even though they can be potentially controlled [10]. Following these considerations, it can be concluded that asthma is a complex chronic disease, whose prevalence crosses all ages. Its clinical features can be observed very early in life, and adolescence represents a very frequent target stage [11]. Thus, one may conclude that the pathogenesis of asthma is set in childhood and adolescence, while, in adulthood, its manifestations are more heterogeneous and dangerous.

Within this framework, the necessity to characterize patients with asthma during both childhood and adolescence emerges, and the study of the microbiota could identify its different roles in inflammatory diseases and asthma in all of its facets.

## 2. The Roles of the Gut Microbiota and Microbiome 

The first descriptions of human-associated microbiota date back to the 1670s–1680s, with the studies conducted by the Dutch microscopist, Antonie van Leeuwenhoek [12]. The first report on microbes in the human system was from Theodor Escherich, an Austrian pediatrician, who observed *Escherichia coli* in the intestinal flora of healthy children and children with gastrointestinal diseases [13]. Subsequent descriptions and isolations of microorganisms from several areas of the human body emerged during the XX century. Although the terms “microbiota” and “microbiome” are often used interchangeably, there are certain differences between them that should be underlined. The term “microbiota” refers to the living microorganisms found in a defined environment (e.g., the oral and gut microbiota). Microbiome refers to the collection of genomes from all the microorganisms in a defined environment, including not only the microbial community, but also microbial structural elements, metabolites, and the environmental context. Thus, the use of the term “microbiome” encompasses a broader spectrum than that defined by the term “microbiota”. Based on the above definitions, we might indicate the microbiome as the dynamic part of the microbiota that is capable of cooperating to organize the core responses of the immune system. A glossary of terms is available in Table 1. The microbiome is diffuse in all parts of the human body, but in this study, we focus on the microorganisms present in the gut and the respiratory system. It is also worth mentioning that our bodies house 10 times more bacterial cells than our total cells and 100 times more genetic material than our genome. With respect to the respiratory system, it is divided into two parts: the upper respiratory tract (URT) and the lower respiratory tract (LRT). Even though there is continuity between the two parts, the anatomical structure is not the same. The corresponding parameters pertaining to these two parts are also different. Moreover, the surface devoted to respiratory exchanges is quite wide, approximately 70 m^2^, and the URT surface is very different. The physiological and physical consequences due to these differences, i.e., pH, pO_2_, pCO_2_, relative humidity (RH), temperature, and density, reflect this situation as well. The presence of different communities of bacteria and viruses, as well as the possible presence of a mycobiota, leads to different impacts according to both their diameter and the reported characteristics [14]. From these points of view, the predominant presence of *Staphylococcus* spp., *Propionibacterium* spp., *Corynebacterium* spp., *Moraxella* spp., and *Streptococcus* spp. has been reported in the nose cavities, while in the trachea and bronchial areas, *Moraxella* spp., *Staphylococcus* spp., *Streptococcus* spp., *Haemophilus* spp., *Rothia* spp., *Veillonella* spp., *Prevotella* spp., and *Leptotrichia* spp. are generally present. Within the lungs, the presence of *Prevotella* spp., *Veillonella* spp., *Streptococcus* spp., and *Trophoderma whipplei* has been reported as well [15]. We can observe that there is not an absolute difference in the composition of the microbiome in different parts of the respiratory system, and the inhalatory phase drives this phenomenon. In the same way, all of the above-mentioned anatomical and physiological features could take part in regulating the whole process. However, the respiratory tract represents the main internal point of contact with the external environment and its microorganisms. The interaction between the environment and the epithelium of the URT and LRT ensures a correct balance to maintain homeostasis [16]. We have to consider that the URT contains a burden of bacteria that is 100 to 10,000 times higher than that of the LRT; therefore, it is likely that the first line of defense and stability is located there; however, it has to be stressed that the microbiome regulates the immune response. The gut provides a comfortable habitat for many microorganisms, and in fact, the gut microbiota is composed of 1000–1200 bacterial species, principally represented by *Firmicutes*, *Bacteroides*, *Proteobacteria*, *Actinobacteria*, and *Fusobacteria* [16]. However, the composition of the gut microbiota varies with internal or external factors, and different people house different compositions of the microbiota and microbiome. The process of aging is another factor that causes changes in the composition of the gut microbiota. In infancy, the gut microbiota plays an essential role in the functions and assessment of the local immune system [17], and the gut microbiome plays an important role in the maturation of local intraepithelial lymphocytes. The gut microbiota also promotes the normal functions of the bowel, such as digestion, decomposition, and the necessary peristalsis, and triggers the production of energy for human metabolism. The energy required by gut microorganisms is produced by short-chain fatty acids (SCFAs) derived from the decomposition of complex carbohydrates caused by the action of *Firmicutes* and *Bacteroides* [18,19]. Acetic acid, propionic acid, and butyric acid also take part in the processes of gluconeogenesis and adipogenesis, regulate the immunological response of the intestines, and inhibit harmful bacterial growth [20]. The gut microbiome also plays a role in the metabolism of bile acids that contribute to gastrointestinal metabolic homeostasis [21,22]. Gut microorganisms regulate the human immune system through their cells and metabolites. It is possible for gut microorganisms to overactivate CD8+T cells and cause chronic inflammation [23], but they are also able to stimulate the synthesis of new dendritic cells [24]. The metabolites of the gut microbiome can induce immune responses to stimulate intestinal chemokine receptors and TNF-alpha production following the stimulation of lipopolysaccharide (LPS) and *Candida albicans*. *Streptococcus parasanguinis* and *Australis* can stimulate interferon-gamma (INF-gamma) production, while *Streptococcus mitis* and *Pneumoniae* take part in IL-1beta production. These findings throw light on the important role of the gut microbiota and the associated microbiome in several fields of medicine, both in balancing the general conditions and in inducing an immunological response in response to particular diseases.

## 3. The Gut–Lung Axis 

It is commonly observed that many patients with asthma or chronic obstructive pulmonary disease (COPD) also suffer from chronic gastrointestinal tract (GIT) diseases [25], and in many cases, there is an association between inflammatory bowel disease (IBD) and inflammatory response at the bronchial and lung level. Despite the different functions of the GIT and respiratory system, they have a common embryonic origin with structural similarity. Therefore, it is not surprising that a functional interaction may exist. In this sense, microbiota crosstalk between these two organs might be assumed, and moreover, the local microbiota can influence immunological responses in distal organs, such as the lungs or brain [24]. Changes in the microbiota caused by aging or external factors, such as diet, diseases, use of antibiotics, or environmental pollution, might also induce different immunological responses in these organs [26]. This has led to the term “gut–lung axis”. An example of this is the appearance of allergic airway diseases after the use of antibiotics in early life [27,28]. The abundance and variability of the microbiota and microbiome depends on environmental conditions, both general and local, and this fact is particularly evident in the gut, where pH, biliary acids, and the digestive process influence defense factors [29]. Considering these factors, it has been observed that, in the gut, only four bacterial phyla are present, namely, *Firmicutes*, *Bacteroides*, *Proteobacteria*, and *Actinobacteria*, but many other microorganisms can be observed when different situations occur [30]. The lungs, on the other side, can show a large surface that can host microorganisms representing a balance among many factors, such as inhalation, exhalation, ingestion, and dropping processes from the nose and other nasal cavities. Owing to these mechanisms, the pulmonary flora can change according to microaspiration and breathing, and the lungs have a lower biomass in comparison with the adjacent sites even though they are involved in continuous air movements that bring in but also remove microorganisms and their particles. This means that dysbiosis in the adjacent sites can possibly affect the lungs. These processes make possible the activation of a disease or a state of phlogosis in the lungs; in this perspective, it is really important to distinguish using independent culture techniques whether the DNA is the expression of viable bacteria [31]. This implies the necessity to strictly observe all the best technical rules during clinical and research investigations.

## 4. Interaction between the Gut and the Lungs

The proper reciprocal interaction between these two organs is the basis for the maintenance of immunological responses to limit the most dangerous phlogistic reactions. As already stressed, the surfaces of these two sites host a great number of microorganisms, which reach their targets via different ways. Firstly, the mucosa represents the first defense line in these two organs, and the microbiota develops many steps of resistance against pathogens. One of the first actions is the inhibition of NFkB, a well-known transcription factor involved in inflammatory reactions and in the production of anti-inflammatory Tregs [32]. These actions are ascribed to non-pathogenic *Salmonella* strains and to the role of *Clostridium* spp. [32]. The same action carried out by Tregs is performed by non-pathogenic species of *Streptococcus pn*. in cooperation with *Haemophilus infl*. In this case, activation of p-38 mitogen protein kinase (MAPK) in toll-like receptors has been described [33,34,35], which elicits a well-coordinated anti-inflammatory action. A demonstration of these actions had been described in premature infants born via caesarean section, where the composition of the microbiota can cause atopic manifestation, and the colonization of *Clostridium difficile* is associated with the onset of an atopic dermatitis phenotype with late development that is representative of a true asthmatic condition [36]. Once again, these and many other data not reported here clearly demonstrate that alterations at the gut level can cause subsequent consequences in the lungs. Another specific and very interesting subject is related to the role of host cells in regulating inflammatory responses and cytokine production. Other examples of the influence of the microbiota on immunity include the ability of *Bacteroides* sp. to elicit the production of Tregs or suppress the TH1/TH2 phenotypes. In this way, it would present a limit to the host inflammatory responses in a strain-specific manner. Moreover, it has been observed that there is limitation of the inflammatory reactions due to metabolic products, such as SCFA and FFA, and the epigenetic regulation of immune cells [37]. According to the above-mentioned findings, it is worth mentioning the main steps of the “hygiene hypothesis and microbiota”. At the end of the 1980s, David Strachan proposed that the decrease in the number of infections during childhood altered the development of the immune system and, therefore, exposed people to an increased risk of allergic diseases [38,39]. The author concluded that early exposure of babies to a farm environment and to contacts with animals and house dust could prevent the onset of allergic diseases, particularly asthma. Such an environment would foster the normal expansion of the microbiota in early infancy; particularly, the dominant phylum of *Actinobacteria* would be the main actor. Contemporary exposure to other infectious agents is associated with remarkable resistance to the development of lung disorders over a lifetime. External factors such as excessive sanitization of the skin, inappropriate use of antibiotics, and improper diet could provoke dysbiosis with subsequent predisposition to future allergic diseases. Moreover, the Old Friends hypothesis, proposed by Graham Rook in 2003 and derived from the Hygiene hypothesis, is based on the assumption that some microorganisms have existed throughout history and have been present during the evolution of the human immune system; in other words, this hypothesis uses an evolutionary framework to identify microbial-driven effects on the immunoregulatory circuits [40]. In short, we co-evolve with microbes, and this interplay affects multiple physiological programs that ultimately impact the development of the effector and regulatory arms of the immune system. Factors that reduce or interrupt exposure to microbial-related signals can induce the onset of chronic inflammatory conditions, such as childhood asthma. Interestingly, pathogen-induced selective pressure on gene expression has been reported, among which are those encoding for T cells, monocytes, NK cells, dendritic cells, and major histocompatibility complex and transcription factors [41]. Probiotics are defined by the FAO/WHO as “live microorganisms which when administered in adequate amounts confer a health benefit on the host” [42]. On the other hand, prebiotics are defined as “a selectively fermented ingredient that allows specific changes, both in the composition and/or activity in the gastrointestinal microflora that confers benefits upon host well-being and health” [43]. In this perspective, as already underlined, the gut and lung dysbiosis documented in children with asthma cooperates to perpetuate the inflammatory reaction; for this reason, it can be considered an actionable target, providing a rationale for the use of probiotics/prebiotics to restore local microbial homeostasis [44]. A list of clinical trials exploring the microbiome as an actionable target in childhood asthma is detailed in Table 2.

**Table 2:** Clinical Trials Focusing on Microbiota and Childhood Asthma. Data was obtained from clinicaltrial.gov by searching with the key terms “microbiome” OR “microbiota” OR “probiotics” OR “prebiotics” AND “asthma” AND “Childhood” OR “adolescence”.

ID Trial	Title	Design	AIM & Target	Status
NCT04527016	Airway Microbiota Based Treatment of Asthma in Preschool Children (AMBT)	Phase 4, single-center, randomized-controlled study	Microbiota phenotype and blood eosinophils level	Completed
NCT05192499	Respiratory Dysbiosis in Preschool Children With Asthma: Predictive of a Severe Form (DREAM)	Exploratory multicentric prospective case-control study.	To find the impact of respiratory dysbiosis and severe asthma	Recruiting
NCT05028153	Azithromycin Treatment of Hospitalized Children With Asthmatic Symptoms (COPSACazt)	Phase 2, double-blind, randomized, controlled clinical trial.	To investigate the effect of a 3-day azithromycin treatment vs placebo in children aged 1–5 years who are hospitalized due to asthma-like symptoms. Airway microbiota, pathogenic bacteria and vira as measured by 16S-rRNA and whole genome sequencing.	Recruiting. Kyvsgaard JN, Ralfkiaer U, Følsgaard N, et al. Azithromycin and high-dose vitamin D for treatment and prevention of asthma-like episodes in hospitalised preschool children: study protocol for a combined double-blind randomised controlled trial. BMJ Open. 2022; 12(4):e054762. doi: 10.1136/bmjopen-2021-054762 [45]
NCT06271213	The Gut-Lung Axis and Respiratory Illness in Children	Observational Model Case-Control Time Perspective Cross-Sectional	Cross-sectional: to investigate correlations between the gut and lung microbiome in 3 cohorts of children aged 0–16 attending the Royal Hospital for Children in Glasgow: Respiratory patients, GI patients and Orthopaedic patients. Longitudinal: Investigating the Gut-Lung axis in asthma pre and post biologics therapy.	Recruiting
NCT04641000	The Alberta BLOOM Long Term Follow Up Study (BLOOM-LTFU)	Prospective, observational clinical cohort study Population: very preterm children (<31 weeks and six days gestation).	To investigate the microbiome alternations resulting from preterm birth and its associations with the risk of immune dysregulation, asthma and allergies.	Terminated
NCT05011071	The Alberta BLOOM Premature Child Study (BLOOM-PCS)	Prospective, observational clinical cohort study Population: 405 premature children (<37 weeks gestation) and their mother/parent/guardian.	To investigate how the microbiome of children develops over the first years of life and its associations with the risk of childhood health outcomes including allergies and asthma. The study will also examine how perinatal factors associate with patterns of microbiome development, and their effects on the microbiome, metabolome and immune development of this population in the first years of life.	Enrolling by invitation
NCT04289441	Probiotics in Paediatric Asthma Management (ProPAM)	Randomized controlled double-blind trial. Population: child allergic asthma and recurring wheezing.	Child undergo probiotic treatment with Bifiasthm with the aim of assessing the reduction in asthma attacks.	Completed Ciprandi G, Schiavetti I, Cioffi L, et al. Probiotics in Pediatric Asthma Management (PROPAM) study: A Post Hoc analysis in allergic children. Ann Allergy Asthma Immunol. 2022; 129(1):111–113. doi: 10.1016/j.anai.2022.04.026. [46]
NCT01366222	Food Concentrates Supplementation to Alleviate Asthma in Children (FSAC)	Allocation: Randomized Interventional: Model Parallel Assignment Masking: Triple (ParticipantCare ProviderOutcomes Assessor) Primary Purpose: Treatment	To determine whether food concentrates supplementation encompassing probiotics are effective to alleviate drug used against symptoms and improve lung function in children with asthma.	Completed

## 5. Asthma and Microbiome

**Figure 1:**
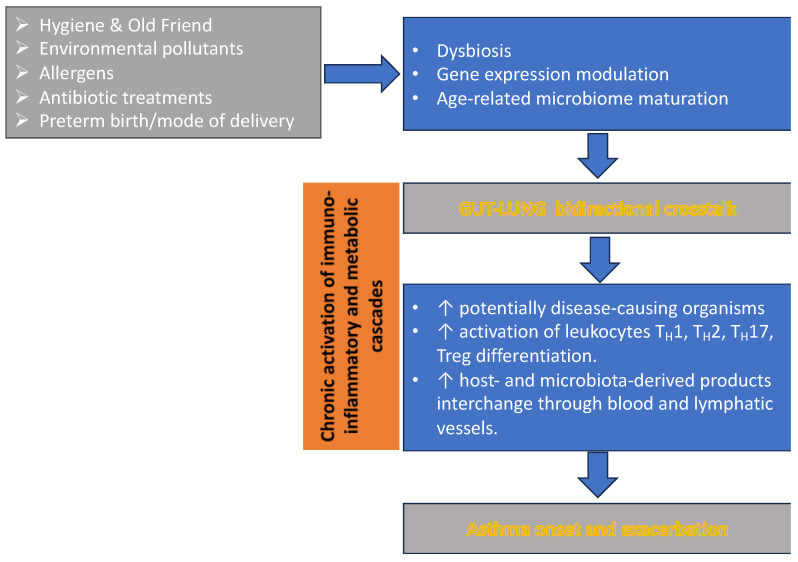
How Microbiota Contributes to the Manifestation of Childhood Asthma. The composition of the lung and gut microbiome and microbiota is influenced by a number of factors, starting from the neonatal and perinatal periods and evolves over the life course. In preterm babies, the low diversity in the composition of the microbiome is associated with a higher relative abundance of asthma-related bacteria (such as Candida and Rhodotorula), which can induce high levels of blood eosinophils and neutrophils and, overall, promote CD4 T-cell dysfunc-tion, which is known to be associated with atopy [63]. Airway colonization with *Streptococ-cus* pneumoniae, *Haemophilus* influenzae, and Moraxella catarrhalis and viral infections (e.g., respiratory syncytial virus and human rhinovirus) are associated with a higher risk of asthma onset during childhood. Thus, dysbiosis of the gut–lung axis [64], in combination with genetic traits and environmental–evolutionary pressure, induces the impairment of im-mune responses and altered immune system.

Asthma is the most frequent non-communicable disease in childhood. A prevalence of 10–15% in children has been reported worldwide [47]. Many risk factors have been identified, including genetic traits, respiratory infections, a suggestive familial anamnesis, air pollution, and an unhealthy lifestyle [48]. The interconnection between asthma and microbiome is reported in Figure 1. Regarding the role of the microbiome in protecting the lungs against asthma and other allergic diseases, it has been reported [49] that the pivotal role of the lungs is similar to the role of the intestines. The lung microbiota might be recognized by pattern recognition receptors (PRRs), and it promotes the production of TH1 lymphocytes from naïve cells that efficiently shift from the TH2 phenotype after birth. In the lungs of neonates, there is a progressive increase in the bacterial load; importantly, there is a shift from the phyla of *Gammaproteobacteria* and *Firmicutes* to that of *Bacteroides*. These bacteria stimulate Tregs that limit or stop the inflammatory reactions in the lungs from infancy to adulthood. This is depicted as a well-balanced situation with a favorable evolution, but in patients with asthma, the observed major prevalence of *Proteobacteria* over *Bacteroides* is associated with lower immune defense [49]. In the field of childhood asthma or atopic diseases, the true difficulty lies in the technical possibility of obtaining reliable samples from the airways or the lungs to gather data that are statistically acceptable. The recourse to oropharyngeal swabs could simplify the procedures, allowing the performance of correct and reliable analysis. It must be remembered that asthma in infancy and childhood is not only very frequent and not simple to detect and manage, but it can be the first step toward the development of COPD in adulthood. Abdel-Aziz M. et al. [50] identified four clusters of children with asthma and other allergic diseases in their study using oropharyngeal swabs. The samples were obtained from the Unbiased Biomarkers for Prediction of Respiratory Disease Outcomes (U-BIOPRED) and analyzed using 16S ribosomal RNA gene sequencing. The Bray–Curtis beta diversity was calculated using these samples. Children with severe asthma were followed for 12–18 months. Through this important methodology, the authors identified four distinct and well-characterized clusters with different clinical features and outcomes. The first cluster was dominated by *Streptococcus* spp., and one or more exacerbations were present in 54% of the cases. Atopic dermatitis was present in 48% of the cases. The second cluster showed a prevalence of *Veillonella*, and exacerbations were more frequent (77%) with a high percentage of atopic dermatitis (78%) in a year. In the third cluster, *Rothia* was dominant, and atopic dermatitis was present in 62% of the cases with a lower percentage of exacerbations (27%) in a year. Finally, the fourth cluster showed the dominant prevalence of *Haemophilus* and *Neisseria*, with 68% of atopic episodes and a percentage of exacerbations of 67% per year. These results are quite interesting and important and allow the identification of some important biomarkers. However, in a complementary analysis, Ghedin E. [51] underscores that the previous authors identified two hallmark pathways that were statistically different in the four clusters: Wnt/Beta-catenin was mainly associated with cluster 4 and TGF-Beta with cluster 2. Both pathways have been reported to be involved in the process of remodeling in chronic asthma. These data demonstrate the importance of studies conducted with samples obtained using oropharyngeal swabs, a less invasive method. The role of commensals that are usually present not just in the lungs or the gut has often been underevaluated, and a study by Rigauts C. et al. [52] demonstrated that a commensal bacterium, *Rothia mucilaginosa*, which is normally present in the oral cavity but also found in the lower airways, inhibits inflammation by acting on the inhibition of NF-kB in chronic asthma as well as many other inflammatory diseases, such as COPD, cystic fibrosis (CF), and bronchiectasis. Its action through the inhibition of NF-kB is extended against other inflammatory and proinflammatory cytokines, including IL-8, IL-1beta, and matrix metalloproteinases, such as (MMP)-1, (MMP)-8, and (MMP)-9 in sputum. This example proves the possibility of a non-specific bacterium to bring its important action in maintaining immunological equilibrium in the respiratory system.

In the introductive section, we have stressed the role of dysbiosis, which can be differently caused, in inducing excessive phlogosis, which might be the basis of asthma. A large-scale meta-analysis [53] demonstrated the association between bacteria colonizing the upper and lower respiratory tracts in young children as a cause of diseases not directly associated with asthma. Particularly, it emphasized the role of *Streptococcus pn*. and *Staphylococcus* Au. and the presence of *Klebsiella pn*. and *Moraxella catarrhalis* in the lower respiratory tract as both commensal and etiological agents of different diseases. In fact, the presence of this kind of flora represents a threat to the optimal immunological balance of the whole respiratory tract. Some considerations on the role exerted by the microbiome of the upper airways should be mentioned, specifically in the nose of schoolchildren, as immediate steps of the natural history of asthma. Yanjiao Zhou et al. [54] collected nasal blow samples within a period of one year in a clinical trial where two time points were foreseen. The aim was to compare the microbiome composition and the control of the disease, considering that all cases were affected by mild-to-moderate persistent asthma controlled with daily inhaled corticoids (ICS). These authors identified a “yellow zone” where the patients expressed signs and symptoms of progression to an exacerbation. As previously reported, it is already known that the microbiome is associated with the severity of this respiratory illness; the novelty of this study lies in its investigation of the role of specific microorganisms in the control or, on the contrary, the risk of both exacerbations and progression toward more serious stages. It had been reported that the predominant presence of the *Corynebacterium + dolosigranulum* genera was associated with a favorable outcome with the lowest rates of YZs, while the presence of *Staphylococcus*, *Streptococcus*, and *Moraxella* was associated with a less favorable outcome. The latter is probably an expression of a bacterial infection able to cause dysbiosis, which forms the basis of an asthmatic exacerbation. In these cases, the likelihood of preventing progression with the use of oral corticoids was estimated as possible. Another interesting point stressed by these authors was the change in airway microbiota from randomization to the YZ. In fact, the bacterial composition changed profoundly, marking a dynamic phenomenon. The authors reported that, during this process, *Streptococcus* was prevalent in the YZ phase, and alpha diversity marked the total bacterial load and total richness during this phase. This process seems particularly impressive when the switching is from *Corynebacterium + dolosigranulum* to *Moraxella* because the worst and most frequent exacerbations are related to the presence of the latter genus. Another interesting point regards the role of viruses. The authors mainly found the presence of human *enterovirus* and *rhinovirus*, but their role did not seem as important as that of *Moraxella*. This study seems important with respect to some points of view. It has been well-known for many years that there is a strong link between bacterial infections and asthma [55], and some bacterial species such as *Staphylococcus aureus*, *Streptococcus pn.*, *Pseudomonas aer.*, *Haemophilus infl*., and *Moraxella catarrhalis* are present during both remission and exacerbations [1]. Atypical bacteria such as *Mycoplasma pn.* and *Chlamydia pn.* can exhibit a similar role [56]. The use of new methods such as 16s ribosomal RNA sequencing has allowed researchers to attain novel and remarkable results in the search for bacterial communities in the gut and the lungs that elicit an active immunological response in both the lungs and other more distant organs. Even if every clinician holds personal classification of asthma according to their everyday activity, Kian Fan Chung [56] clearly summarizes the many types of asthma in their classification. Following the topic of this work, it seems useful to stress the correlations between the microbiota and asthma. As previously pointed out, in mild asthma, there is a high prevalence of *Proteobacteria*, and this flora is associated with a worse clinical presentation and an overexpression of Th17-related genes [57]. An association between *Firmicutes* and *Actinobacteria* has been observed in more severe cases [58], while that between *Firmicutes* and *Trophoderma* is present in eosinophilic asthma [59]. Neutrophilic asthma is marked by an association between *H. influenzae* and *M. catarrhalis* [60]. During the first year of life, it has been observed that the presence of some bacteria, such as *Haemophilus*, *Streptococcus*, *Moraxella*, and *Staphylococcus*, is predictive of the emergence of asthma during infancy. Bringing up babies in a farm environment might be protective (the Hygiene hypothesis), as previously reported; however, the condition of neonatal dysbiosis is associated with recurrent wheezing in childhood. Therefore, these data demonstrate that appropriate bacterial colonization in neonates predisposes them to have strong defense mechanisms against asthma and allergy [56]. It is important to come back to the role of viruses, which often elicit asthmatic exacerbations. In healthy individuals, rhinovirus does not form part of the composition of the microbiome, but in individuals with COPD, its presence leads to a remarkable presence of proteobacteria, which plays a role in eliciting exacerbations [61], and persistent bacterial colonization in the lower airways might be favorable to viral infections that are able to maintain a persistent inflammatory condition [62].

## 6. Azithromycin and Wheezing in Children

The role of antibiotics has been viewed with caution in the field of microbiota and microbiome. This is fitting, considering their possibility in inducing dysbiosis if employed inappropriately. However, azithromycin has proved to have a positive impact on episodes of wheezing, cough, and breathlessness, and its relationship with the microbiome composition seems rather well-founded [65]. Its potential anti-inflammatory and antimicrobial effects cannot be ignored. Thorsen et al. [66] analyzed the possible relationship between the nasopharyngeal microbiome and asthma-like symptoms in children aged 12–36 months after being treated with azithromycin. The sample was randomized to receive either azithromycin (10 mg/kg/day for three days) or placebo. In the arm of azithromycin, the authors observed a shorter duration of symptoms in 63.3% of cases; moreover, using 16S rRNA obtained from hypopharyngeal aspirate at the beginning of the study, they could better analyze their data. The most frequent isolates were *Moraxella*, *Haemophilus*, *Streptococcus*, and *Neisseria*, and the increased richness of the bacterial composition was associated with a greater response to azithromycin. Particularly, the presence of *Veillonella*, *Leuconostoc*, and *Vibrio* was associated with an increased effect, while *Neisseria* led to a less impressive action. This study [66] underlines the undoubtable antibacterial action of azithromycin and its greater effect in the presence of higher richness and abundance of the microbiome in hypopharyngeal samples. On the other hand, the presence of *Prevotella* seems to be associated with a longer duration of episodes due to the lower activity of azithromycin [66]. The work is really important because the action of a macrolide drug is demonstrated to be useful with an immediate outcome, and these results might indicate that its early use presents a preventive measure to limit the future onset of mild or severe asthma. Unfortunately, to date, there are no data indicating the effect of azithromycin on the microbiome after treatment. A second limitation regards the use of hypopharyngeal sample, which probably does not represent the precise composition of the microbiome in the lower airways.

## 7. Green Living

A current opinion asserts that green living in infancy might play a role in preventing childhood asthma. The reasons include an escape from negative environmental factors, such as air pollution and exposure to excessive heat. On one hand, the possibility of spending more time outdoors with physical activity and encountering a variety of microbiota is considered positive [67]. On the other hand, it has been observed that early exposure to aeroallergens and pollens is associated with the possibility of an increase in wheezing and the development of asthma [68]. In order to clarify these contrasting opinions, Stanescu et al. [69] conducted a retrospective study with children born in Toronto, Canada, from April 2006 to March 2007, following a methodology that also took into account their mothers’ exposure during pregnancy and considered the role of three canopy areas where these children could be exposed. The model built by these researchers is rather complex but very interesting, and it has led to some intriguing results. Children of mothers who were exposed to pollens during pregnancy had an increased risk of asthma, especially with increased exposure to weed pollen. This negative effect was registered if exposure occurred in the later part of the first trimester or at the beginning of the first trimester. A protective effect of the tree canopy area on babies and children was found, but this effect progressively disappeared with exposure to increased weed pollen concentrations in childhood and adolescence. Exposure during the first year of life to total pollen concentration was associated with an increased risk of asthma, and the negative effect observed in the first three years was mainly attributed to a high weed concentration. The protective effect of the tree canopy was observed mainly when birth occurred during fall. These results are interesting and present a contradictory scenario, but the authors underscore that living in green space can enrich contact with a variety of microbiotas, and this might lead to better resistance to allergy and asthma. The conclusion is that early exposure to pollens might increase the risk of developing asthma, but tree canopies offer at least partial protection.

## 8. Conclusions

In conclusion of this brief report, we must recognize that the link between asthma and the gut and lung microbiota is very tight, even if not yet totally explored. Asthma is defined as a heterogeneous disease based on chronic inflammation, and this condition is influenced by the interplay of many elements, including the microbiome and microbiota. We must remember that the gut microbiome as well as the microbiome located in the lungs and in the upper and lower airways can elicit both “defensive” phlogosis and immunological stimulation, which helps to balance the response triggered by asthma and other atopic diseases. Asthma also exhibits clinical phenotypes that are often different from each other, and the study of specific microbiomes might be a milestone in elucidating those pathogenetic elements that support these different phenotypes. Unfortunately, despite the early works on the microbiota being produced twenty years ago, an organic perspective and longitudinal studies are lacking. The methods of investigations allow for in-depth study of the composition of the microbiome and its wide actions in the immunological field, and it would be possible to connect the steps that support certain situations in a comprehensive network. A subsequent point for further investigation is the role of prebiotics and probiotics. At present, we are looking at a wide use of probiotics that are not always clinically justified, and it has to be remembered that probiotics have been defined as live organisms that, when administered in adequate amounts, can provide health benefits to the host. However, probiotics are like common drugs with possible side effects. Therefore, it is very important to acquire adequate knowledge on their correct use. This aim will be reached when we have complete knowledge and competence in the use of probiotics. The role of the microbiome in asthma, particularly during infancy, childhood, and adolescence, will be seen in the future as pivotal for the control of asthma from both the clinical and social points of view. 

## Figures and Tables

**Table 1:** Microbiota or Microbiome? These two scientific terms are not synonyms, and many other terms deserve a deeper understanding.

Term	Definition
Alpha Diversity	different types of sequences in a sample.
Amplicon	an amplified fragment of DNA deriving from a marker gene generated by PCR
Beta Diversity	different types of sequences commonly shared in samples
Dysbiosis	when the normal structure of Microbiome is disturbed by external cause such as diseases or use of antibiotics.
Metabolomics	assessment of the metabolites in a given sample that leads to the metabolically active microrganisms.
Metagenomics	the study of the whole genome of a group of miscrorganisms assessing the potentiality of their functions.
Microbiome	The totality of microbes with their genes that are harbored by the Microbiota and the milieu in which they interact”
Microbiota	All the microbes that are found in a particular region or habitat
Otu (Operational Taxonomic Unit)	specific sequences based on sequence similarity to reference genes
Resistome	antibiotic resistance genes in a community.
Richness	number of taxa in a single population
Sequencing	technique allowing also millions of DNA sequences obtained from a single sample
Taxon	a group of phylogenetically connected microbes belonging to the same taxonomic group i.e., order, family, genus
Taxonomy	the science devoted to identify different species and classify them
Virome	all the Viruses present in an environment.

## Data Availability

Not applicable.
